# An idiopathic left internal iliac artery aneurysm in an 11-year-old boy: a case report

**DOI:** 10.1186/s13256-025-05050-3

**Published:** 2025-02-06

**Authors:** Meghdad Ghasemi Gorji, Amirhossein Baratinezhad, Rojan Abdollahzadeh Mirali

**Affiliations:** 1 Department of Vascular Surgery, Shiraz University of Medical Science, Shiraz, Iran; 2 Student Research Committee, Shiraz University of Medical Sciences, Shiraz, Iran; 3 Shiraz University of Medical Sciences, Shiraz, Iran

**Keywords:** Idiopathic aneurysm, Pediatric aneurysm, Iliac artery aneurysm, Vascular surgery, Case report

## Abstract

**Background:**

Idiopathic isolated aneurysms in pediatric patients are rare and present significant diagnostic and therapeutic challenges owing to their low incidence, small anatomical size, and the associated risks of injury to surrounding structures. Timely diagnosis and the selection of the most appropriate intervention require careful consideration.

**Case presentation:**

An 11-year-old Persian boy presenting with abdominal pain for 2 months before admission was diagnosed with an isolated left internal iliac artery aneurysm through computed tomography imaging. He underwent open surgery for excision of the aneurysm. The iliac vein was adherent to the aneurysmal sac and significantly compressed, complicating the dissection and exploration. Nevertheless, proximal control was achieved, and the iliac vein was gradually separated and decompressed, allowing for the safe excision of the aneurysmal sac. The surgery was completed without intraoperative or postoperative complications.

**Conclusion:**

Successful surgical intervention in pediatric patients with iliac artery aneurysms is achievable with careful planning and technique. This case report demonstrates the effectiveness of surgical management, with the patient remaining asymptomatic and showing no signs of recurrence during follow-up.

## Background

Arterial aneurysms in children are exceedingly rare, with isolated common iliac artery aneurysms even more uncommon [1]. While most pediatric arterial aneurysms are attributed to underlying causes, such as trauma or collagen vascular diseases, idiopathic aneurysms represent an exceptionally rare etiology [2].

Similar case reports indicate that such aneurysms pose significant diagnostic and therapeutic challenges, with high operative mortality rates reported in literature [3]. The subtle presentation and lack of specific symptoms make early detection difficult, underscoring the importance of maintaining a high index of suspicion in pediatric patients [4].

This report presents the case of an 11-year-old boy diagnosed with a symptomatic left internal iliac artery aneurysm. The case offers insights into the diagnostic workup and surgical treatment, contributing to the understanding of idiopathic aneurysms in children and highlighting the need for continued research and awareness in this underrepresented area of vascular surgery.

## Case presentation

**Fig. 1:**
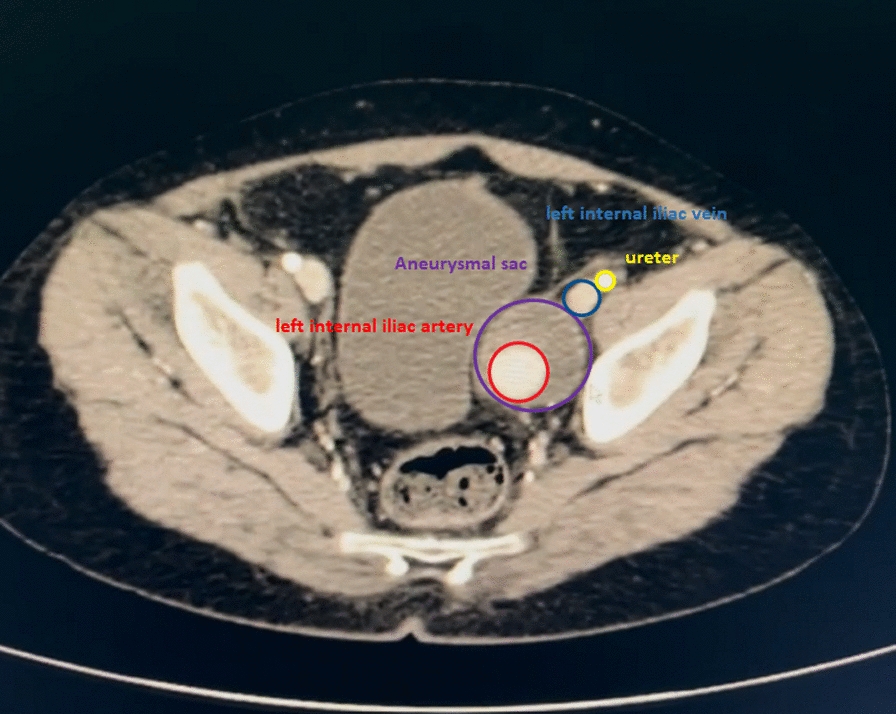
The figure shows a transverse view of anatomical structure near the honorary small sac, including the left internal iliac artery, left internal iliac vein, and ureter

**Fig. 2:**
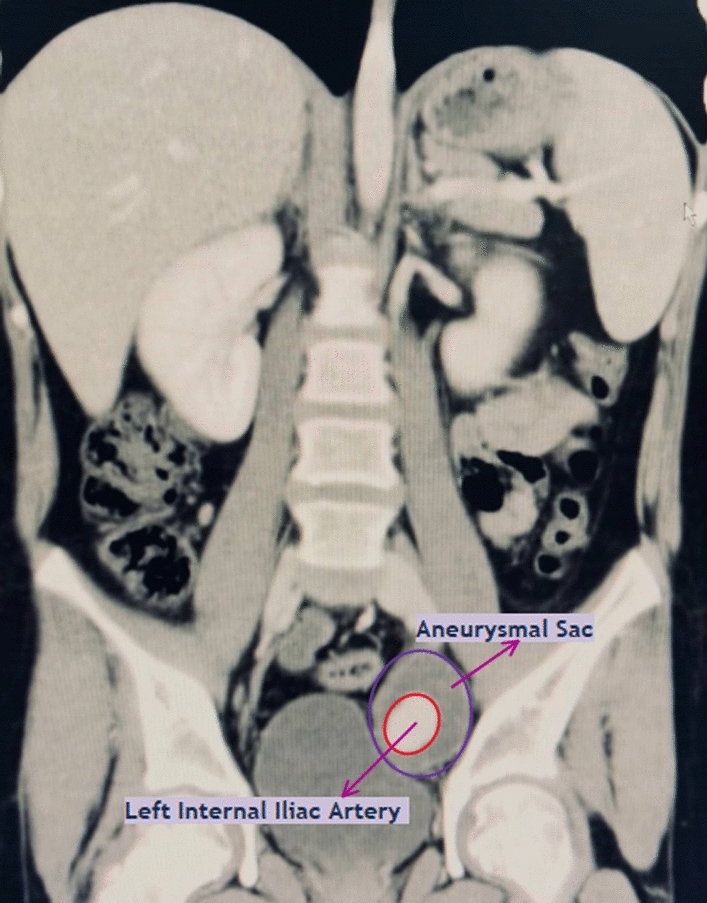
This figure shows a coronal view of the left internal iliac artery within the aneurysmal sac, demonstrating compression on the bladder. Arrows are pointing to the aneurysmal sac and the left internal iliac artery

An 11-year-old Persian boy, previously healthy, presented with severe abdominal pain 2 months before admission. Initial investigations revealed no abnormalities, including a complete blood count (CBC), urinalysis, and sonography. Persistent pain prompted further evaluation, and a computed tomography (CT) scan identified a 4 cm × 4 cm aneurysm of the left internal iliac artery. Owing to the critical nature of the condition, surgical intervention was deemed necessary. Preoperative CT angiography confirmed the diagnosis and aided in surgical planning (Figs. 1 and 2).

During surgery, the patient was placed under general anesthesia with a combination of agents: isoflurane (250 ml solution) for maintenance, lidocaine 2% (5 ml ampule) for local infiltration, and morphine (10 mg ampule) for pain management. Additional medications included atracurium (50 mg/5 ml ampule) for intubation, fentanyl citrate (0.5 mg/10 ml ampule) for analgesia, midazolam (5 mg ampule) for sedation, thiopental (Nesdonal, 1 g ampule) for induction, and neostigmine (0.5 mg ampule) to reverse muscle relaxants post-surgery.

Surgical intervention began with a low midline laparotomy incision. Owing to the patient’s small size, careful dissection was required in the limited working space, with meticulous attention to avoid injury to adjacent structures, particularly the ureter. Dissection of the iliac artery and vein was complicated by the adherence of the iliac vein to the aneurysmal sac and significant compression, making it difficult to distinguish from surrounding tissues.

**Fig. 3:**
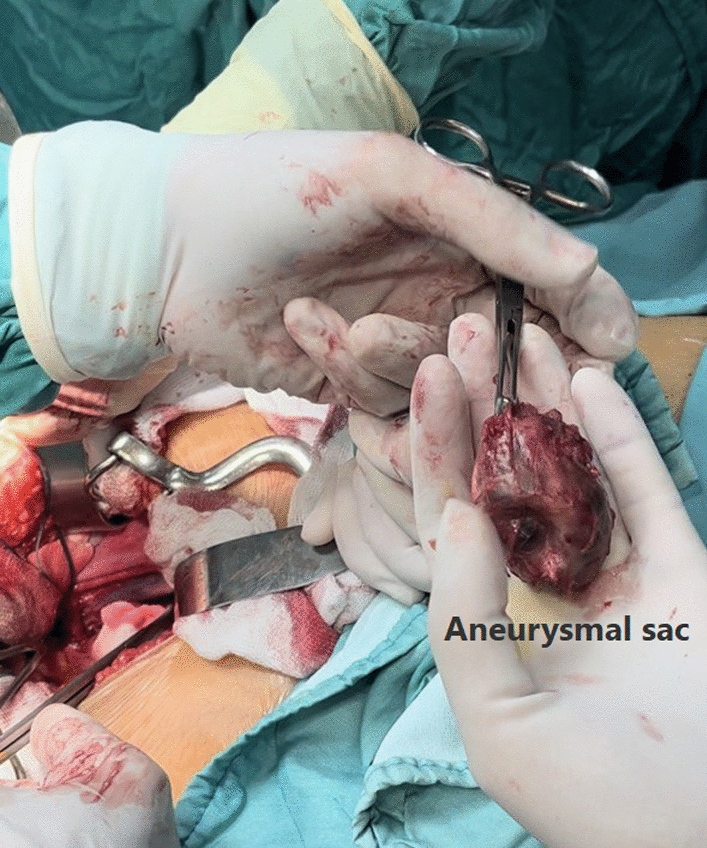
The aneurysmal sac removed completely

After separating the vein and artery from the aneurysmal sac, proximal control was established over the right common iliac artery, followed by the right internal and external iliac arteries. A weight-adjusted dose of 2500 units of heparin was administered prior to arterial clamping. Given the absence of post-procedural bleeding, no reversal agent was required. The aneurysmal branch of the left internal iliac artery was ligated, and the aneurysm was excised (Fig. 3). Hemostasis was achieved using Surgicel, and the aneurysm cavity and retroperitoneum were closed with Vicryl 2–0 sutures. The abdominal wall was closed with Vicryl 1 sutures, and the skin was sutured with Nylon 3–0. A sterile dressing was applied.

The excised aneurysmal sac was sent for pathology, confirming a true aneurysm with evidence of thrombosis. Further work-up revealed no evidence of infection, and tests for tumor markers and autoantibodies (including antinuclear antibodies, double-stranded DNA, antineutrophil cytoplasmic antibodies, and antiphospholipid antibodies) were negative. After ruling out other potential causes, the diagnosis was confirmed as an idiopathic aneurysm.

Postoperative follow-up included abdominopelvic abdominal sonography, which revealed a standard liver size and parenchymal echogenicity; a well-distended gall bladder without stones; and normal-sized spleen, pancreas, and kidneys. A hypoechoic structure measuring approximately 33 mm × 34 mm posterior to the urinary bladder, suggesting hematoma formation, warranted further imaging for evaluation. No free fluid was detected in the abdominal cavity.

The patient’s stable postoperative course, without signs of complications or hematoma in subsequent evaluations, supports the patient’s recovery. Planned additional imaging aims to ensure a complete recovery and further assessment.

## Discussion

Diagnosing an internal iliac artery aneurysm in pediatric patients is particularly challenging owing to its rarity and often asymptomatic nature [3]. Literature indicates that idiopathic aneurysms in children are often detected incidentally during evaluation for unrelated symptoms, with a notable absence of apparent predisposing factors [1, 2, 5, 6].

Imaging studies, especially CTA, are crucial for aneurysm diagnosis, with other options including magnetic resonance angiography (MRA) and Doppler ultrasound for peripheral aneurysms [7]. Given the rarity of iliac artery aneurysms in children, they are frequently overlooked, making timely diagnosis essential in this population. Studies show that pediatric aneurysms are uncommon, occurring in less than 0.2% of cases [8]. Increased awareness and consideration of this diagnosis in pediatric patients, particularly with imaging studies, can be life saving.

Managing pediatric idiopathic aneurysms is challenging owing to the condition’s rarity and unique anatomical considerations [2]. In this case, surgical intervention was chosen as the primary treatment, aligning with recommendations in literature [2, 9]. This decision was influenced by the natural progression of such aneurysms, which often lead to rupture, thrombosis, or thromboembolism if left untreated [4].

For the 11-year-old patient with an internal iliac artery aneurysm, open surgery was preferred over endovascular procedures owing to the patient’s young age and small vascular anatomy, which posed challenges for safe device deployment [10]. Furthermore, considering long-term durability and the potential need for future interventions, open surgery provided a more definitive solution, reducing the likelihood of reinterventions compared with endovascular methods [10–12].

On the basis of available literature, we have compiled a table of key findings related to pediatric internal iliac artery aneurysms (IIAAs) and their management, concluding our discussion:

**Table:** 

Aspect	Details
Incidence	Pediatric aneurysms are rare, occurring in < 0.2% of cases. IIAAs in children are even rarer, often underreported owing to asymptomatic presentation
Diagnostic challenges	Diagnosing IIAAs in children is difficult owing to their rarity and nonspecific symptoms. Imaging (CTA, MRA, Doppler) is essential
Primary symptoms	Symptoms often include abdominal pain or are incidentally found during imaging for unrelated conditions
Management approach	Open surgery is preferred in young patients owing to anatomical challenges and long-term durability concerns. Endovascular repair is less suitable for small vascular anatomy
Prognosis	Timely surgical intervention prevents complications such as rupture, thrombosis, or embolism. Post-surgery outcomes are generally favorable with no immediate complications if conducted meticulously
Considerations	It is necessary to monitor for recurrence or complications. Awareness among clinicians is crucial for earlier diagnosis and better outcomes

Our surgical approach involved meticulous dissection and complete excision of the aneurysmal sac, aligning with contemporary methods reported by Krupski *et al*. [3]. Direct operative repair was associated with lower mortality and morbidity rates; in this case, no intraoperative bleeding or damage to surrounding structures occurred.

## Conclusion

Timely diagnosis of iliac artery aneurysms in pediatric patients is critical, as delayed detection can lead to life-threatening rupture and mortality. The rarity of these aneurysms often results in missed diagnoses, emphasizing the need for increased awareness and careful evaluation. Managing aneurysms in this population requires a tailored approach, considering unique anatomical and physiological factors. Specifically, surgical management must consider the proximity of vital adjacent structures, which pose additional risks during dissection and repair.

## Data Availability

You may contact the corresponding author for further case information if interested. You may also visit www.vascularsurgery.ir for more detailed videos and photos of this case.

## Abbreviations

CBC: Complete blood count
CTA: Computed tomography angiography
MRA: Magnetic resonance angiography
